# Latent profile analysis of psychological capital and its association with quality of life in colorectal cancer patients undergoing chemotherapy

**DOI:** 10.3389/fpubh.2026.1923920

**Published:** 2026-10-28

**Authors:** Qiqi He, Jiuqun Li, Xiaohang Su

**Affiliations:** 1 Department of Nursing, Yangtze University, Jingzhou, Hubei, China; 2 Department of Nursing, Peking University Shenzhen Hospital, Shenzhen, Guangdong, China; 3 School of Nursing and Health Sciences, Henan University, Kaifeng, Henan, China

**Keywords:** chemotherapy, colorectal cancer, latent profile analysis, psychological capital, quality of life

## Abstract

**Objective:**

To identify latent profiles of psychological capital (PsyCap) among colorectal cancer patients undergoing chemotherapy, compare demographic and clinical characteristics across profiles, and examine the association between PsyCap profiles and quality of life.

**Methods:**

A convenience sampling method was used to recruit 327 colorectal cancer patients undergoing chemotherapy at Peking University Shenzhen Hospital from January to May 2026. Data were collected using a general information questionnaire, the Psychological Capital Questionnaire (PCQ-26), and the Functional Assessment of Cancer Therapy-Colorectal (FACT-C). Latent profile analysis identified PsyCap profiles. Chi-square tests were used to compare demographic and disease-related characteristics across latent classes. One-way ANOVA was used when variances were homogeneous. Homogeneity of variance was assessed using Levene’s test. When the assumption of homogeneity of variance was violated, Welch’s ANOVA was used for overall group comparisons, followed by Games–Howell *post hoc* tests for pairwise comparisons. Eta-squared (η^2^) was calculated as an omnibus effect size for comparisons of FACT-C scores across PsyCap profiles. Multiple linear regression was used to analyze the association between PsyCap latent profiles and quality of life.

**Results:**

Three latent profiles of PsyCap were identified among colorectal cancer patients undergoing chemotherapy: Low PsyCap (35.5%), Moderate PsyCap (40.1%), and High PsyCap (24.5%). Significant differences were observed among the three profiles in all FACT-C domains and the total score (all Welch *p* < 0.001), with higher PsyCap profiles showing higher quality-of-life scores. After adjusting for demographic and clinical characteristics, PsyCap profiles remained significantly associated with FACT-C total scores. Compared with the Low PsyCap profile, the Moderate PsyCap profile (*B* = 7.073, 95% CI: 3.077–11.070, *p* = 0.001) and High PsyCap profile (*B* = 13.740, 95% CI: 9.096–18.384, *p* < 0.001) had higher FACT-C total scores.

**Conclusion:**

PsyCap among colorectal cancer patients undergoing chemotherapy showed heterogeneity primarily in overall level and was classified into three sample-relative profiles: Low, Moderate, and High PsyCap. Higher PsyCap profiles were associated with better quality of life after adjustment for demographic and clinical characteristics. The person-centered classification may complement continuous PsyCap scores by identifying subgroups with different overall levels of psychological resources. Early identification of patients with relatively low PsyCap may help inform individualized supportive strategies.

## Introduction

Colorectal cancer is one of the most common malignant tumors in the world. In recent years, both its incidence and mortality have increased in different countries. By 2040, the global burden of colorectal cancer is expected to increase by 63%, with approximately 3.2 million new cases (1). Chemotherapy is one of the major treatment modalities for colorectal cancer, including neoadjuvant chemotherapy, adjuvant chemotherapy, and palliative treatment (2). During chemotherapy, patients frequently experience concurrent symptoms such as fatigue, diarrhea, abdominal distension, and sleep disturbance (3–5). These experiences can easily lead to negative emotions, including anxiety, depression and stigma, which severely impair patients’ mental health and quality of life (6, 7). Quality of life is therefore an important outcome in patients with CRC undergoing chemotherapy, reflecting the multidimensional impact of disease and treatment on physical, emotional, social/family, and functional well-being. The FACT-C is particularly appropriate for this population because it integrates general domains of cancer-related quality of life with colorectal cancer-specific concerns, thereby capturing both broad functional well-being and disease-specific experiences. Beyond clinical and treatment-related factors, positive psychological resources may also be associated with QoL. Previous studies have linked resilience and self-efficacy with better QoL among patients with CRC (8, 9).

Psychological capital (PsyCap) describes a positive psychological state that individuals exhibit during growth and development (10). It comprises four components: self-efficacy, resilience, hope and optimism (11). These factors not only help individuals cope with stress but also enhance their adaptability and capacity for recovery when facing difficulties (12). PsyCap is an important indicator of patients’ mental health. For colorectal cancer patients undergoing chemotherapy, a high level of psychological capital can help maintain a positive mental state, stimulate patients’ initiative and engagement during treatment and rehabilitation, and serve as a key internal resource that supports them in coping with treatment-related adverse effects, maintaining a positive attitude, and strengthening confidence in recovery. Therefore, higher PsyCap levels have been associated with better psychological adaptation and quality of life in cancer populations (13–15).

Previous studies have shown that the level of PsyCap among patients with cancer remains insufficient. However, most existing studies have used variable-centered approaches to evaluate PsyCap based on the total score or dimension scores. Although such approaches can reveal overall relationships among variables, they are unable to identify potential heterogeneity within a population. In fact, because patients differ in disease severity, treatment experience, social environment and psychological adjustment capacity, their PsyCap may nevertheless show meaningful heterogeneity across individuals that cannot be fully characterized by a single sample-level mean. Therefore, a person-centered approach is needed to further explore the latent heterogeneity of PsyCap among colorectal cancer patients undergoing chemotherapy.

The LPA is a widely used person-centered classification method. Based on a latent variable model, it identifies unobserved latent subgroups according to individuals’ response patterns across multiple continuous variables. Individuals within the same profile share similar characteristics, whereas individuals across different profiles exhibit clear differences. Unlike traditional variable-centered methods such as regression analysis and factor analysis, LPA focuses on classifying individuals rather than analyzing overall associations among variables. Traditional variable-centered approaches usually assume that participants are relatively homogeneous and primarily examine associations among variables in the overall sample; therefore, they may overlook the presence of distinct subgroups. This may limit their explanatory power when psychological characteristics show substantial individual differences. By contrast, LPA can identify heterogeneity within a population and is therefore more suitable for exploring latent types of patients’ psychological characteristics. In recent years, an increasing number of LPA-based studies have found significant individual differences in psychosocial characteristics among patients with cancer, including social isolation, self-regulatory fatigue and resilience (16–18). These findings suggest that applying LPA to investigate latent profiles of PsyCap among colorectal cancer patients undergoing chemotherapy has both theoretical and practical value.

Importantly, previous person-centered studies have identified heterogeneous PsyCap profiles in other clinical populations, including patients with breast cancer and ischemic stroke (19, 20). However, these findings cannot directly establish whether similar PsyCap heterogeneity exists among patients with CRC undergoing chemotherapy. To our knowledge, evidence regarding the latent profiles of PsyCap in this specific population remains limited, and it is still unclear whether different PsyCap profiles are associated with differences in CRC-specific QoL. Thus, examining PsyCap from a person-centered perspective may complement conventional total-score approaches by providing additional information on psychological heterogeneity within this population. Therefore, this study is aimed to identify latent profiles of PsyCap among colorectal cancer patients undergoing chemotherapy using LPA, and analyze factors associated with different classes, further examine differences in quality of life among patients with different PsyCap classes and the relationship between these classes and overall quality of life. The findings may provide evidence to inform stratified psychological assessment and supportive care for colorectal cancer patients undergoing chemotherapy.

## Methods

### Study design and participants

This cross-sectional study used convenience sampling to recruit colorectal cancer patients undergoing chemotherapy at Peking University Shenzhen Hospital from January to May 2026. The inclusion criteria were as follows: (1) pathologically diagnosed primary colon cancer or rectal cancer; (2) currently receiving chemotherapy; (3) age ≥ 18 years; (4) awareness of their disease condition; (5) ability to correctly understand the questionnaire and cooperate with the survey. The exclusion criteria were as follows: (1) other severe diseases, such as severe cardiovascular disease; (2) mental disorders or psychological illnesses that prevented completion of the questionnaire. According to Kendall (21), the sample size should be at least 5 to 10 times the number of variables considered. In this study, 26 variables were considered. Allowing for a 20% rate of invalid responses, the required sample size was *n* = (26 × 10)/(1–0.2) = 325. In addition, simulation studies of latent profile analysis have shown that sample sizes of 250, 500 and 1,000 are commonly used and can provide adequate statistical power under appropriate class-separation conditions (22). A total of 350 questionnaires were distributed. After excluding 23 questionnaires because they showed highly repetitive or patterned response patterns and were therefore considered invalid, 327 valid questionnaires were included in the final analysis, yielding an effective response rate of 93.4%. This study was reviewed and approved by the Medical Ethics Committee of Peking University Shenzhen Hospital (approval number: 2026–019).

### Data collection

Before the survey, all investigators received standardized training. Prior to questionnaire administration, investigators used standardized instructions to explain the study purpose and questionnaire completion requirements to participants. After written informed consent was obtained, paper-based questionnaires were administered individually. Participants completed the questionnaires independently whenever possible. For participants who were unable to complete the questionnaires independently, trained investigators recorded their verbatim responses without interpretation or prompting. Assisted completion was not documented as a separate variable during data collection; therefore, the exact number of questionnaires completed with investigator assistance could not be determined retrospectively. After completion, questionnaires were checked on site for missing responses or unclear entries, which were verified with participants when possible. No missing PCQ-26 item responses remained in the final analytic sample of 327 participants. All questionnaires were collected on site, and the data were independently checked and entered by two researchers.

### Measures

#### Sociodemographic and clinical characteristics

A self-designed questionnaire was used to collect sociodemographic and disease-related information. It included 17 items: age, sex, marital status, educational level, place of residence, smoking history, comorbid chronic diseases, employment status, medical insurance type, family per capita monthly income, disease type, time since diagnosis, pathological stage, treatment modality, stoma status, chemotherapy regimen and number of chemotherapy cycles.

#### Psychological capital

PsyCap was assessed using the Psychological Capital Questionnaire (PCQ-26) developed by Zhang et al. (23). The PCQ-26 consists of four dimensions: self-efficacy (items 1, 3, 5, 7, 9, 11, and 13), resilience (items 2, 4, 6, 8, 10, 12, and 14), hope (items 15, 17, 19, 21, 23, and 25), and optimism (items 16, 18, 20, 22, 24, and 26). Each item is rated on a 7-point Likert scale ranging from 1 (strongly disagree) to 7 (strongly agree). Items 8, 10, 12, 14, and 25 were reverse-scored according to the original scoring instructions. Dimension scores were calculated as the mean of the corresponding items, and the overall PsyCap score was calculated as the mean of all 26 items. Higher scores indicate higher levels of PsyCap. The PCQ-26 has demonstrated good reliability and validity among Chinese populations. In the present study, Cronbach’s α coefficients were 0.956 for the total scale and 0.860, 0.864, 0.789, and 0.842 for self-efficacy, resilience, hope, and optimism, respectively.

### Quality of life

Quality of life was measured using the Functional Assessment of Cancer Therapy-Colorectal (FACT-C), developed by Ward et al. (24), and translated into Chinese by Yang et al. (25). The FACT-C consists of 36 items covering five domains: physical well-being (PWB), social/family well-being (SWB), emotional well-being (EWB), functional well-being (FWB), and colorectal cancer-specific concerns (CCS). Items are rated on a 5-point scale from 0 (not at all) to 4 (very much), with negatively worded items (GP1–GP7, GE1, GE3–GE6, C1, C2, and C5) reverse-scored. The CCS contains nine items; C1–C7 contribute to the scored CCS, whereas the ostomy-specific items C8 and C9 are not included in the CCS or FACT-C total score. The CCS score ranges from 0 to 28, and the FACT-C total score, calculated as the sum of PWB, SWB, EWB, FWB, and CCS, ranges from 0 to 136, with higher scores indicating better quality of life. When eligible item responses were missing, domain scores were prorated according to the FACIT scoring guidelines by multiplying the sum of completed item scores by the total number of items in the domain and dividing by the number of items answered. In the present sample, GS7 was answered by only five participants and was therefore excluded from the internal consistency analysis. Cronbach’s α was 0.966 for the 33 items included in the reliability analysis; the α coefficients for PWB, SWB (based on GS1–GS6), EWB, FWB, and CCS were 0.917, 0.678, 0.897, 0.890, and 0.761, respectively.

### Statistical analysis

Data were analyzed using IBM SPSS Statistics 26.0 and Mplus 8.3. Categorical variables were expressed as frequencies and percentages [*n* (%)], and continuous variables were expressed as means ± standard deviations. Harman’s single-factor test was used to assess common method bias, and Cronbach’s alpha was used to evaluate internal consistency. The 26 items of the Psychological Capital Questionnaire were used as observed indicators for LPA. Models with one to five classes were fitted sequentially, and the optimal model was determined comprehensively based on the Akaike information criterion (AIC), Bayesian information criterion (BIC), sample-size adjusted Bayesian information criterion (aBIC), entropy, Lo–Mendell–Rubin likelihood ratio test (LMR), and bootstrap likelihood ratio test (BLRT). To avoid local maxima, 1,000 random sets of starting values were specified in the LPA, and 250 sets were retained for final-stage optimization. The best log-likelihood value for the final model was replicated. Model-estimated class proportions were reported for model comparison, whereas participants were assigned to their most likely latent profile for descriptive analyses and subsequent between-profile comparisons. Chi-square tests were used to compare categorical variables across PsyCap latent profiles. For continuous variables, homogeneity of variance was assessed using Levene’s test. One-way analysis of variance was used when the homogeneity assumption was met; when this assumption was violated, Welch’s ANOVA was used, followed by Games–Howell *post hoc* tests for pairwise comparisons. Eta-squared (*η^2^*) was calculated as an omnibus effect size for comparisons of FACT-C scores across PsyCap profiles. Comparisons of demographic and clinical characteristics across PsyCap profiles were considered exploratory analyses. Multiple linear regression analysis was performed to examine the association between PsyCap latent profiles and quality of life. Covariates were selected *a priori* based on clinical relevance and previous literature, including age, educational level, comorbidity status, disease stage, treatment modality, and stoma status (7, 9). These variables were selected because they may influence patients’ psychological adaptation and quality of life during chemotherapy. Multicollinearity was assessed using tolerance and variance inflation factor (VIF). The assumptions of multiple linear regression were further assessed by examining the distribution of residuals, standardized residual-versus-predicted plots, and influential observations using standardized residuals, leverage values, and Cook’s distance. All statistical tests were two-sided, and *p* < 0.05 was considered statistically significant.

## Results

### Common method bias and multicollinearity tests

As all data were collected using self-report questionnaires, Harman’s single-factor test was performed to assess common method bias. Ten factors with eigenvalues greater than 1 were extracted, and the first factor explained 36.68% of the variance, which was below the recommended threshold of 40% (26). Therefore, no dominant single factor was identified.

To support the predictive analyses, multicollinearity diagnostics were performed for the observed variables. The tolerance values ranged from 0.288 to 0.910, and the variance inflation factor (VIF) values ranged from 1.098 to 3.473, indicating no evidence of problematic multicollinearity.

### Descriptive statistics

A total of 327 colorectal cancer patients undergoing chemotherapy were included in this study. The mean age was 58.75 years (SD = 10.65; range, 26–85 years). Among them, 60.6% were male, 58.4% were aged 45–65 years, 89.3% were married, 67.0% lived in urban areas, 49.8% had a junior high school education or below, 42.2% were employed and 52.9% had a family per capita monthly income of 5,000–10,000 yuan. Disease-related characteristics showed that 64.5% were diagnosed with colon cancer, 56.6% had been diagnosed for more than 6 months, 48.9% were at stage IV, 64.2% received surgery combined with chemotherapy, 12.8% had a stoma and 47.1% had received more than six chemotherapy cycles. The sociodemographic and disease-related characteristics of the patients are shown in Table 1.

**Table 1:** Characteristics of patients across PsyCap profiles (*N* = 327).

Variables	Category	*n*(%)	Profile 1 (*n* = 116)	Profile 2 (*n* = 131)	Profile 3 (*n* = 80)	*χ^2^*	*P*
Age(years)						11.371	0.023
	<45	35(10.7)	5(4.3)	21(16.0)	9(11.3)		
	45–65	191(58.4)	78(67.2)	71(54.2)	42(52.5)		
	>65	101(30.9)	33(28.4)	39(29.8)	29(36.3)		
Sex						0.900	0.638
	Male	198(60.6)	68(58.6)	78(59.5)	52(65.0)		
	Female	129(39.4)	48(41.4)	53(40.5)	28(35.0)		
Marital status						4.504	0.105
	Married	292(89.3)	109(94.0)	115(87.8)	68(85.0)		
	Unmarried/divorced/widowed	35(10.7)	7(6.0)	16(12.2)	12(15.0)		
Educational						14.209	0.007
	Junior high school or below	163(49.8)	68(58.6)	66(50.4)	29(36.3)		
	Senior high school/technical secondary school	67(20.5)	23(19.8)	29(22.1)	15(18.8)		
	Junior college or above	97(29.7)	25(21.6)	36(27.5)	36(45.0)		
Place of residence						0.962	0.618
	Rural	108(33.0)	41(35.3)	44(33.6)	23(28.8)		
	Urban	219(67.0)	75(64.7)	87(66.4)	57(71.3)		
Smoking						0.363	0.834
	Yes	94(28.7)	35(30.2)	38(29.0)	21(26.3)		
	No	233(71.3)	81(69.8)	93(71.0)	59(73.8)		
Comorbid chronic disease						3.014	0.222
	Yes	173(52.9)	62(53.4)	75(57.3)	36(45.0)		
	No	154(47.1)	54(46.6)	56(42.7)	44(55.0)		
Employment status						8.907	0.063
	Employed	138(42.2)	49(42.2)	57(43.5)	32(40.0)		
	No fixed occupation	110(33.6)	46(39.7)	44(33.6)	20(25.0)		
	Retired	79(24.2)	21(18.1)	30(22.9)	28(35.0)		
Medical insurance type						4.950	0.084
	Employee medical insurance	141(43.1)	45(38.8)	53(40.5)	43(53.8)		
	Resident medical insurance	186(56.9)	71(61.2)	78(59.5)	37(46.3)		
Family per capita monthly income						8.192	0.085
	<5,000	118(36.1)	52(44.8)	43(32.8)	23(28.8)		
	5,000–10,000	173(52.9)	54(46.6)	75(57.3)	44(55.0)		
	>10,000	36(11.0)	10(8.6)	13(9.9)	13(16.3)		
Disease type						0.395	0.821
	Colon cancer	211(64.5)	77(66.4)	82(62.6)	52(65.0)		
	Rectal cancer	116(35.5)	39(33.6)	49(37.4)	28(35.0)		
Time since diagnosis (month)						1.806	0.771
	<1	25(7.6)	11(9.5)	7(5.3)	7(8.8)		
	1–6	117(35.8)	41(35.3)	49(37.4)	27(33.8)		
	>6	185(56.6)	64(55.2)	75(57.3)	46(57.5)		
Disease stage						2.191	0.701
	I–II	29(8.9)	12(10.3)	10(7.6)	7(8.8)		
	III	138(42.2)	44(37.9)	56(42.7)	38(47.5)		
	IV	160(48.9)	60(51.7)	65(49.6)	35(43.8)		
Treatment modality						1.781	0.939
	Chemotherapy	80(24.5)	31(26.7)	30(22.9)	19(23.8)		
	Surgery + chemotherapy	210(64.2)	70(60.3)	88(67.2)	52(65.0)		
	Surgery + chemotherapy + radiotherapy	23(7.0)	9(7.8)	9(6.9)	5(6.3)		
	Chemotherapy + radiotherapy	14(4.3)	6(5.2)	4(3.1)	4(5.0)		
Stoma						11.010	0.004
	Yes	42(12.8)	23(19.8)	16(12.2)	3(3.8)		
	No	285(87.2)	93(80.2)	115(87.8)	77(96.3)		
Chemotherapy regimen						3.120	0.538
	XELOX regimen	144(44.0)	54(46.6)	55(42.0)	35(43.8)		
	FOLFOX/FOLFOXIRI regimen	34(10.4)	10(8.6)	12(9.2)	12(15.0)		
	Other	149(45.6)	52(44.8)	64(48.9)	33(41.3)		
Number of chemotherapy cycles						1.261	0.868
	<3	93(28.4)	36(31.0)	34(26.0)	23(28.8)		
	3–6	80(24.5)	25(21.6)	35(26.7)	20(25.0)		
	>6	154(47.1)	55(47.4)	62(47.3)	37(46.3)		

Profile 1 = low PsyCap profile; Profile 2 = moderate PsyCap profile; Profile 3 = high PsyCap profile. Categorical variables are presented as *n* (%).

### Latent profile analysis

Table 2 presents the model fit indices for the latent profile models. Although the LPA was conducted using the 26 PCQ items as observed indicators, the profiles were plotted using the mean scores of the four PsyCap dimensions to facilitate interpretation. Models with one to five latent profiles were fitted sequentially. The fit indices provided competing evidence regarding the optimal number of profiles. Although AIC and aBIC continued to decrease with additional profiles, BIC reached its minimum for the four-profile solution, and the BLRT remained significant for both the four- and five-profile solutions. In contrast, the LMR test was significant for the three-profile solution but not for the four-profile solution (*p* = 0.449), indicating that the addition of a fourth profile did not significantly improve model fit according to this criterion. The three-profile solution also showed slightly higher classification accuracy (entropy = 0.933) than the four-profile solution (entropy = 0.921). Examination of the competing four-profile solution further indicated that the additional profile primarily represented a finer subdivision of overall PsyCap level, with the four dimensions remaining broadly parallel rather than forming a qualitatively distinct configuration (Supplementary Table S2 and Supplementary Figure S1). Considering the competing statistical evidence, classification quality, parsimony, and substantive interpretability, the three-profile solution was retained.

**Table 2:** Comparison of model fit indices for latent profiles of PsyCap.

Model	AIC	BIC	aBIC	Entropy	LMR	BLRT	Model-estimated class proportions (%)
1	23906.429	24103.507	23938.566	–	–	–	100.00
2	20897.285	21196.692	20946.107	0.969	<0.001	<0.001	62.25/37.75
3	20156.959	20558.695	20222.468	0.933	0.0002	<0.001	35.10/40.62/24.28
4	19862.519	20366.583	19944.713	0.921	0.4494	<0.001	22.60/37.73/25.66/14.00
5	19766.195	20372.588	19865.075	0.912	0.2339	<0.001	20.10/33.11/21.83/14.60/10.36

AIC, Akaike information criterion; BIC, Bayesian information criterion; aBIC, adjusted Bayesian information criterion; LMR, Lo–Mendell–Rubin; BLRT, Bootstrap likelihood ratio test. Class proportions were based on the model-estimated posterior probabilities and may differ slightly from proportions calculated according to individuals’ most likely latent profile membership.

The three latent profiles of PsyCap are shown in Figure 1. Based on the most likely latent profile membership, 116 (35.5%), 131 (40.1%), and 80 (24.5%) participants were classified into Profiles 1, 2, and 3, respectively. The overall PsyCap score was 4.19 ± 0.69 in the total sample, and the mean scores were 3.50 ± 0.26, 4.22 ± 0.24, and 5.16 ± 0.32 in the Low PsyCap, Moderate PsyCap, and High PsyCap profiles, respectively. Profile 1 showed the lowest scores across all four PsyCap dimensions and was labeled the Low PsyCap profile; Profile 2 showed intermediate scores and was labeled the Moderate PsyCap profile; and Profile 3 showed the highest scores and was labeled the High PsyCap profile. Dimension-specific scores are presented in Supplementary Table S1.

**Figure 1:**
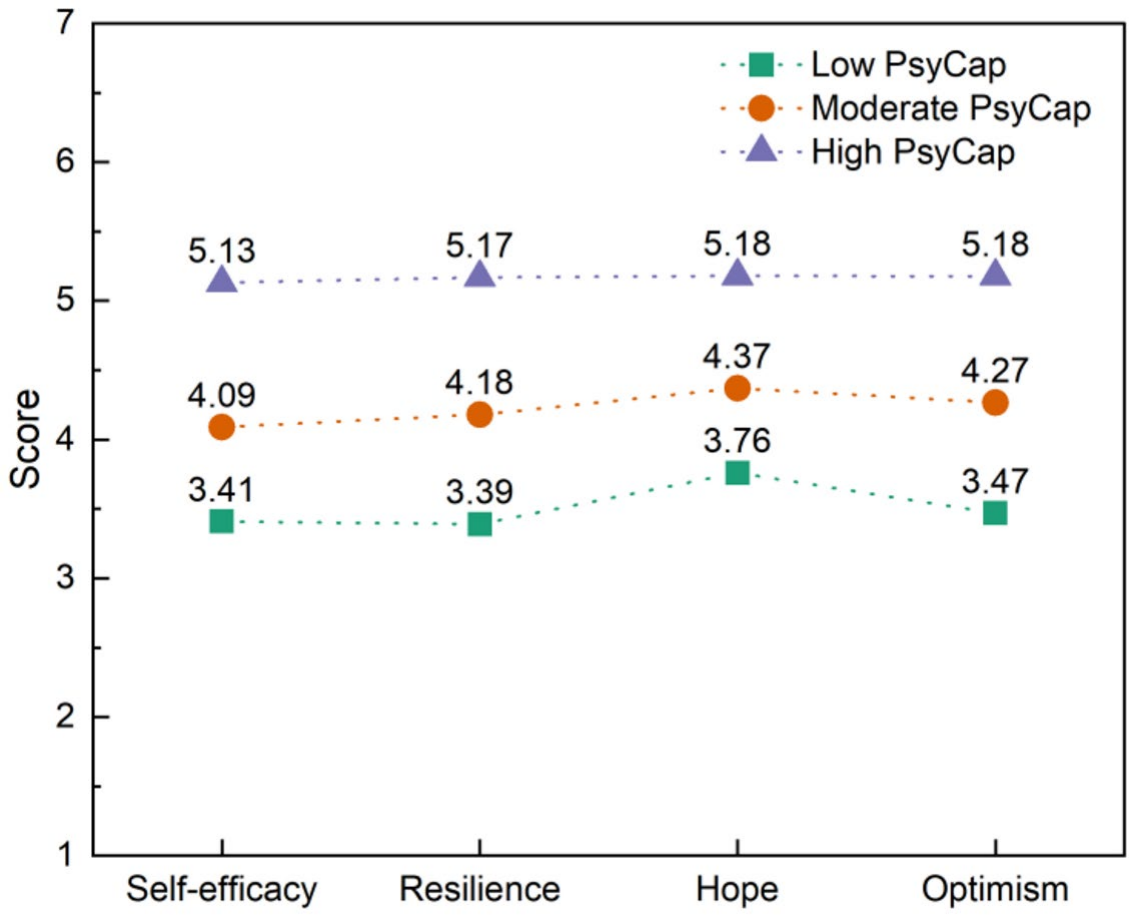
Latent profiles of PsyCap among colorectal cancer patients undergoing chemotherapy. The *y*-axis represents the mean scores of the four PsyCap dimensions, and *x*-axis represents the 4 dimensions of PsyCap.

### Differences in quality of life across PsyCap latent profiles

As shown in Table 3, Levene’s tests indicated heterogeneity of variances for all FACT-C domains and the total score (all *p* < 0.05). Therefore, Welch’s ANOVA and Games–Howell *post hoc* tests were applied. Significant differences were observed across the three PsyCap profiles in all FACT-C domains and the total score (all Welch *p* < 0.001), with *η^2^* values ranging from 0.098 to 0.122. Games–Howell *post hoc* comparisons further showed that the High PsyCap profile had significantly higher scores than the Moderate PsyCap profile, which in turn had significantly higher scores than the Low PsyCap profile across all FACT-C domains and the total score (all pairwise *p* < 0.05).

**Table 3:** Comparison of quality-of-life scores among colorectal cancer patients undergoing chemotherapy across PsyCap latent profiles.

Variable	Low PsyCap	Moderate PsyCap	High PsyCap	Welch F	*P*	*η^2^*	Comparison
PWB	11.22 ± 3.61	12.73 ± 4.72	15.11 ± 4.83	19.098	<0.001	0.103	P3 > P2 > P1
SWB	18.07 ± 2.46	19.10 ± 3.00	20.62 ± 3.49	16.464	<0.001	0.098	P3 > P2 > P1
EWB	12.38 ± 3.21	13.62 ± 3.85	15.66 ± 4.07	18.378	<0.001	0.104	P3 > P2 > P1
FWB	14.20 ± 3.29	16.16 ± 4.22	18.08 ± 4.55	23.412	<0.001	0.122	P3 > P2 > P1
CCS	16.70 ± 3.00	17.95 ± 3.27	19.70 ± 3.83	17.702	<0.001	0.106	P3 > P2 > P1
FACT-C	72.57 ± 13.86	79.55 ± 17.89	89.17 ± 19.61	22.257	<0.001	0.122	P3 > P2 > P1

*Post hoc* pairwise comparisons were performed using the Games-Howell test. PWB = physical well-being; SWB = social/family well-being; EWB = emotional well-being; FWB = functional well-being; CCS = colorectal cancer subscale; FACT-C = Functional Assessment of Cancer Therapy-Colorectal. *η*^2^ represents the omnibus effect size.

### Multiple linear regression of PsyCap latent profiles and quality of life

Multiple linear regression analysis was performed to examine the association between PsyCap latent profiles and quality of life. Based on clinical relevance and previous literature, age, educational level, comorbidity status, disease stage, treatment modality, and stoma status were included as covariates. The overall regression model was statistically significant [*F*(13, 313) = 10.292, *p* < 0.001] and explained 29.9% of the variance in FACT-C total scores (*R^2^* = 0.299, adjusted *R^2^* = 0.270). Diagnostic analyses did not indicate major violations of the regression assumptions. Standardized residuals ranged from −2.359 to 2.474, the maximum Cook’s distance was 0.030, and visual inspection of the residual histogram, normal P–P plot, and standardized residual-versus-predicted plot did not indicate major departures from residual distribution or homoscedasticity assumptions.

After adjusting for demographic and disease-related characteristics, PsyCap latent profiles remained significantly associated with quality of life. Compared with patients in the low PsyCap profile, patients in the moderate PsyCap profile (*B* = 7.073, 95% CI: 3.077–11.070, *p* = 0.001) and high PsyCap profile (*B* = 13.740, 95% CI: 9.096–18.384, *p* < 0.001) reported significantly higher FACT-C total scores. These findings indicated that PsyCap profiles remained significantly associated with quality of life after adjustment for the selected demographic and clinical covariates (Table 4).

**Table 4:** Multiple linear regression analysis of factors associated with quality of life among colorectal cancer patients undergoing chemotherapy.

Variable	*B*	*SE*	*β*	*t*	*P*	95% CI
Age						
>65	Ref					
45–65	−7.203	1.956	−0.196	−3.683	<0.001	−11.051, −3.355
<45	−20.296	3.244	−0.347	−6.256	<0.001	−26.679, −13.913
Education						
Junior college or above	Ref					
Senior high school/technical secondary school	−1.591	2.497	−0.035	−0.637	0.524	−6.503, 3.321
Junior high school or below	−4.717	2.060	−0.130	−2.290	0.023	−8.770, −0.664
Comorbidity						
No	Ref					
Yes	−2.239	1.798	−0.062	−1.245	0.214	−5.777, 1.298
TNM stage						
I–II	Ref					
III	−6.355	3.188	−0.173	−1.993	0.047	−12.627, −0.083
IV	−2.936	3.193	−0.081	−0.920	0.358	−9.218, 3.345
Treatment modality						
Chemotherapy alone	Ref					
Surgery + chemotherapy	6.780	2.168	0.180	3.127	0.002	2.514, 11.046
Surgery + chemotherapy + radiotherapy	7.152	3.809	0.101	1.878	0.061	−0.342, 14.646
Chemotherapy + radiotherapy	3.721	4.573	0.042	0.814	0.416	−5.277, 12.719
Stoma						
No	Ref					
Yes	−13.202	2.707	−0.244	−4.878	<0.001	−18.528, −7.877
PsyCap profiles						
Low PsyCap	Ref					
Moderate PsyCap	7.073	2.031	0.191	3.482	0.001	3.077, 11.070
High PsyCap	13.740	2.360	0.326	5.821	<0.001	9.096, 18.384

*R*^2^ = 0.299, adjusted *R*^2^ = 0.270, *F*(13, 313) = 10.292, *p* < 0.001. Ref = reference category.

## Discussion

Based on the LPA results, this study identified three latent profiles of PsyCap among colorectal cancer patients undergoing chemotherapy: the low PsyCap profile, the moderate PsyCap profile, and the high PsyCap profile. These labels reflect relative differences in PsyCap within the present sample rather than established clinical categories or cut-off values. These findings suggest heterogeneity in PsyCap levels within this population. The Moderate PsyCap profile accounted for the largest proportion (40.1%), whereas the High PsyCap profile represented the smallest proportion (24.5%), indicating that the largest subgroup of patients was characterized by an intermediate level of PsyCap relative to the other identified profiles. This may be related to the multiple stressors that colorectal cancer patients face during chemotherapy, including the threat of disease, treatment-related adverse reactions, limitations in role functioning and uncertainty about the future. The present findings are generally consistent with studies of patients with breast cancer and ischemic stroke, suggesting that heterogeneity in PsyCap may also be observed across different clinical populations (19, 20). Unlike continuous-score approaches that primarily describe overall levels, LPA identifies subgroups characterized by different overall levels of PsyCap while also allowing the configuration of individual PsyCap dimensions to be examined. Although the identified profiles were primarily differentiated by overall PsyCap level rather than qualitatively distinct dimensional configurations, LPA provides an empirical, model-based classification rather than relying on arbitrary cut-off points of the total PCQ-26 score. This person-centered approach may therefore provide additional information for identifying subgroups with different levels of psychological resources and may facilitate more individualized psychological assessment and supportive care.

As shown in Figure 1 and Supplementary Table S1, the three latent profiles were primarily distinguished by their overall levels of PsyCap, while displaying broadly similar patterns across the four dimensions. Hope tended to be among the higher-scoring dimensions across profiles; however, differences among the four dimensions were generally modest, particularly in the High PsyCap profile. Overall, this pattern further suggests that the identified profiles primarily reflect quantitative differences in psychological resources rather than qualitatively distinct dimensional configurations.

This study further found significant differences in quality of life among patients with different PsyCap latent profiles. Importantly, after adjusting for clinically relevant demographic and disease-related characteristics, PsyCap profiles remained significantly associated with FACT-C total scores. The regression model explained 29.9% of the variance in quality of life, suggesting that PsyCap profiles may represent a relevant psychological correlate of quality of life after accounting for the selected demographic and clinical covariates. This finding is consistent with previous studies reporting associations between positive psychological resources and quality of life among colorectal cancer survivors and broader cancer populations (14, 15). As a developable and malleable positive psychological resource, PsyCap may be associated with more positive perceptions and appraisals of disease-related experiences (12). Patients with higher PsyCap may also report greater confidence in disease management and treatment, a greater tendency to find positive meaning in their illness experiences, and more optimistic expectations regarding future treatment outcomes; these characteristics may be associated with more favorable psychological adjustment (27, 28). In addition, higher PsyCap has been associated with more positive coping behaviors, greater treatment engagement, and better psychological adaptation to disease-related challenges (29–31). Previous intervention research has reported improvements in psychological resources and well-being-related outcomes following positive psychological interventions (32). Higher PsyCap has also been associated with more favorable psychological outcomes, including lower levels of stress, fatigue, depression, and anxiety (33–36). Therefore, the present findings suggest that higher PsyCap profiles are associated with better quality of life, while previous evidence indicates that cognitive appraisal, coping capacity, and adaptation to disease-related stress may provide possible contexts for understanding this association. However, these potential explanatory pathways were not directly examined in the present study and should therefore be interpreted as possible explanations rather than established mechanisms.

The findings of this study suggest that PsyCap among colorectal cancer patients undergoing chemotherapy is clearly heterogeneous and that quality of life differs significantly across PsyCap latent profiles. Therefore, clinical healthcare professionals should pay attention to the assessment of patients’ PsyCap, especially the psychological needs and adaptation status of patients with low PsyCap. Patients in the Low PsyCap profile may warrant greater attention during psychological assessment and supportive care. These findings support the potential clinical relevance of assessing PsyCap in supportive care; however, whether interventions targeting PsyCap can improve quality of life requires prospective evaluation.

### Limitations

This study has several limitations. First, the cross-sectional design precludes determination of the temporal or causal relationship between PsyCap and quality of life, and reverse causality cannot be excluded; patients with better quality of life may also report higher levels of PsyCap. Although several clinically relevant demographic and disease-related factors were adjusted for in the regression analysis, residual confounding from unmeasured factors may remain. Future longitudinal studies are needed to clarify the temporal relationship between PsyCap and quality of life. Second, the sample was recruited from a single medical center, and the generalizability of the findings should be further validated in different regions and larger samples. Finally, data were collected using self-report scales, which may be affected by social desirability bias and information bias. Future research should incorporate objective clinical indicators and longitudinal follow-up designs to clarify the temporal relationships and potential mechanisms linking PsyCap with health outcomes in patients with colorectal cancer.

## Conclusion

The findings suggest that heterogeneity in PsyCap among colorectal cancer patients undergoing chemotherapy was expressed primarily as differences in overall PsyCap level rather than qualitatively distinct dimensional configurations. Three sample-relative profiles—Low, Moderate, and High PsyCap—were identified. Higher PsyCap profiles were associated with better quality of life after adjustment for demographic and clinical characteristics. These findings suggest that person-centered classification may complement continuous PsyCap assessment by identifying subgroups with different overall levels of psychological resources.

## Data Availability

The raw data supporting the conclusions of this article will be made available by the authors, without undue reservation.

## References

[1] Morgan E Arnold M Gini A Lorenzoni V Cabasag CJ Laversanne M . Global burden of colorectal cancer in 2020 and 2040: incidence and mortality estimates from GLOBOCAN. Gut. (2023) 72:338–44. doi: 10.1136/gutjnl-2022-327736, 36604116

[2] General Office of National Health Commission of the People’s Republic of China, Oncology Branch of the Chinese Medical Association. The standard for diagnosis and treatment of Chinese colorectal cancer (2023 version). Med J Peking Union Med Coll Hosp. (2023) 14:706–33. doi: 10.12290/xhyxzz.2023-0315

[3] Zhang J He Q Wang M Lv X Mao D Li J . Core symptoms in patients with colorectal cancer receiving chemotherapy: a network analysis. Support Care Cancer. (2025) 33:532. doi: 10.1007/s00520-025-09600-9, 40461707

[4] Kawasaki Y Kakimoto K Tanaka Y Shimizu H Nishida K Numa K . Relationship between chemotherapy-induced diarrhea and intestinal microbiome composition. Digestion. (2023) 104:357–69. doi: 10.1159/000528282, 37231829 PMC10614279

[5] Tang WW Han ML Xu SH Deng YX Shen Q. Analysis of sleep quality, disease uncertainty, and psychological tolerance in patients undergoing chemotherapy for digestive tract malignancies. World J Clin Cases. (2024) 12:4247–55. doi: 10.12998/wjcc.v12.i20.4247, 39015909 PMC11235551

[6] Chamsi A Ezzaairi F Sahli J Belaid I Ammar N Bourigua R . Assessment of depression, anxiety and fatigue in Tunisian patients in recovery from colon cancer and their impact on quality of life. J Cancer Res Clin Oncol. (2023) 149:4269–74. doi: 10.1007/s00432-022-04334-1, 36064985 PMC9444694

[7] Siddiqui MT Shaukat F Khan MR Zahid N Arbani S. Quality of life of colorectal cancer patients and its association with anxiety and depression: cross-sectional study at a tertiary care hospital in low middle income country. J Surg Res. (2024) 301:336–44. doi: 10.1016/j.jss.2024.06.015, 39018953

[8] Tamura S. Factors related to resilience, anxiety/depression, and quality of life in patients with colorectal cancer undergoing chemotherapy in Japan. Asia Pac J Oncol Nurs. (2021) 8:393–402. doi: 10.4103/apjon.apjon-2099, 34159232 PMC8186384

[9] Lee Y Kim E. Effect size of factors influencing colorectal cancer survivors' quality of life: a systematic review and meta-analysis. Cancer Med. (2026) 15:e71841. doi: 10.1002/cam4.71841, 42036776 PMC13111402

[10] Luthans F Avolio BJ Avey JB Norman SM. Positive psychological capital: measurement and relationship with performance and satisfaction. Pers Psychol. (2007) 60:541–72. doi: 10.1111/j.1744-6570.2007.00083.x

[11] Zhang L Wider W Fauzi MA Jiang L Tanucan JCM Naces Udang L. Psychological capital research in HEIs: bibliometric analysis of current and future trends. Heliyon. (2024) 10:e26607. doi: 10.1016/j.heliyon.2024.e26607, 38404889 PMC10884929

[12] Zhao D Li Y Zhou M Wang Q Li G Qi M . The effect of mindfulness and psychological capital on mental health of breast cancer patients: based on polynomial regression and response surface analysis. J Psychosoc Oncol. (2024) 42:190–207. doi: 10.1080/07347332.2023.2233951 37435866

[13] Yang D She T Gui G Li L Zhou Z Liu L . Psychological capital and death anxiety in pancreatic cancer patients: a latent profile analysis. Front Psychiatry. (2025) 16:1627422. doi: 10.3389/fpsyt.2025.1627422 PMC12457670 41000353

[14] Sheikh-Wu SF Anglade D Gattamorta K Downs CA. Relationships between colorectal cancer survivors' positive psychology, symptoms, and quality of life. Clin Nurs Res. (2023) 32:171–84. doi: 10.1177/10547738221113385, 35996878

[15] Guo YF Wang KF Cross W Lam L Plummer V Li J. Quality of life in cancer patients with different preferences for nurse spiritual therapeutics: the role of psychological capital. J Adv Nurs. (2022) 78:991–1000. doi: 10.1111/jan.15023, 34423462

[16] Liao Y Liu X Wu X Li C Li Y. Social isolation profiles and conditional process analysis among postoperative enterostomy patients with colorectal cancer. BMC Psychol. (2024) 12:782. doi: 10.1186/s40359-024-02304-5 PMC11670519 39722073

[17] Zhou L Xiao T Zheng Y Liu C Ma Q Li F . Profiles and influencing factors for self-regulatory fatigue in patients with colorectal cancer. Nurs Res. (2026) 75:96–104. doi: 10.1097/NNR.0000000000000879 41452785

[18] Howard Sharp KM Tillery Webster R Cook J Okado Y Long A Phipps S. Profiles of resilience, distress, and posttraumatic growth in parents of children with cancer and the relation to subsequent parenting and family functioning. J Pediatr Psychol. (2023) 48:375–85. doi: 10.1093/jpepsy/jsac097, 36668686 PMC10118855

[19] Zhang Y Liu Z Wang X Gu Y. Influencing factors associated with psychological capital among ischemic stroke patients: a latent profile analysis. Psychol Res Behav Manag. (2024) 17:4043–52. doi: 10.2147/prbm.s482943, 39619944 PMC11608031

[20] Yin Y Zhang YW Yan TT Hu MY Chen XJ. Latent profiles analysis and the influencing factors of psychological capital in breast cancer patients. J Nurs Sci. (2025) 40:72–6. doi: 10.3870/j.issn.1001-4152.2025.04.072

[21] Ni P Chen JL Liu N. Sample size estimation in quantitative nursing research. Chin J Nurs. (2010) 45:378–80. doi: 10.3761/j.issn.0254-1769.2010.04.037

[22] Tein JY Coxe S Cham H. Statistical power to detect the correct number of classes in latent profile analysis. Struct Equ Model Multidiscip J. (2013) 20:640–57. doi: 10.1080/10705511.2013.824781, 24489457 PMC3904803

[23] Zhang K Zhang S Dong YH. Positive psychological capital: measurement and relationship with mental health. Stud Psychol Behav. (2010) 8:58–64.

[24] Ward WL Hahn EA Mo F Hernandez L Tulsky DS Cella D. Reliability and validity of the functional assessment of Cancer therapy-colorectal (FACT-C) quality of life instrument. Qual Life Res. (1999) 8:181–95. doi: 10.1023/a:1008821826499, 10472150

[25] Yang Z Lu YB Li YF Wan CH Luo JH Meng Q . Evaluation of the functional assessment of Cancer therapy-colorectal (V4.0) in Chinese version. Chin J Tissue Eng Res Clin Rehabil. (2007) 11:8753–6.

[26] Podsakoff PM MacKenzie SB Lee JY Podsakoff NP. Common method biases in behavioral research: a critical review of the literature and recommended remedies. J Appl Psychol. (2003) 88:879–903. doi: 10.1037/0021-9010.88.5.879, 14516251

[27] Mohsenipouya H Motallebi Z Mousavinasab N Sangani A Roy N Mamun MA. Subjective well-being among Iranian breast cancer patients: exploring the influential role of psychological capital. J Psychosoc Oncol. (2025) 43:248–64. doi: 10.1080/07347332.2024.2381546, 39052985

[28] Guo S Cui P Wang P Liu W Shao M Li T . The chain mediating effects of psychological capital and illness perception on the association between social support and acceptance of illness among Chinese breast cancer patients: a cross-sectional study. Eur J Oncol Nurs. (2025) 75:102800. doi: 10.1016/j.ejon.2025.102800, 39914132

[29] Tan M Wu GF Wu SJ Feng L Tang XX Hu H. Mediating role of psychological capital in perceived social support and self-management in HIV/AIDS patients. J Nurs (China). (2026) 33:54–7. doi: 10.16460/j.issn2097-6569.2026.02.054

[30] He Y Wang YJ Xie J Miao JN. Analysis on the mediating effect of psychological capital on perceived social support and patient activation among stroke patients in the recovery period. J Nurs (China). (2023) 30:65–70. doi: 10.16460/j.issn1008-9969.2023.07.065

[31] Li N Han RR Xu CX Sun YQ Jiao GM. The mediating effect of psychological capital between social limitation and cancer adaptation in elderly patients with breast cancer. Mod Prev Med. (2023) 50:2396–401.

[32] Jin Y Wang Y Ma H Tian X Wang H. The efficacy and mechanism of positive psychological intervention on well-being for colostomy patients: a randomized controlled trial. Support Care Cancer. (2022) 30:5747–57. doi: 10.1007/s00520-022-06951-5, 35332374

[33] Guo YQ Zhao B Xu Y. Mediating effect of psychological capital between perceived stress and anxiety symptoms among primary caregivers of stroke patients. Chin J Prev Control Chronic Dis. (2024) 32:111–4. doi: 10.16386/j.cjpccd.issn.1004-6194.2024.02.007

[34] Liu LX Zhou HM Zang TZ Tian XQ Fan XX Feng ZJ . Latent profile analysis of psychological capital and antenatal depression among pregnant women. Zhong Nan Da Xue Xue Bao Yi Xue Ban. (2025) 50:2255–66. doi: 10.11817/j.issn.1672-7347.2025.250438, 42032987 PMC12989417

[35] Aman HK. The differential effect of sources of social support on postpartum depression: the mediating role of resilience. Philos Ethics Humanit Med. (2025) 20:35. doi: 10.1186/s13010-025-00190-2, 41131562 PMC12551333

[36] Pourshahriar H Khalili B Shokri O Fatehi F. The assessment of the perceived stress and the quality of life in the patients with myasthenia gravis: the mediating role of the psychological capital and social support. Curr J Neurol. (2024) 23:124–30. doi: 10.18502/cjn.v23i2.16841, 39744654 PMC11685554

