# Supplementary material for: Latent profile analysis of psychological capital and its association with quality of life in colorectal cancer patients undergoing chemotherapy
Source: Front Public Health. 2026 Oct 28;14:1923920. doi: 10.3389/fpubh.2026.1923920 (PMC13751523; doi:10.3389/fpubh.2026.1923920)
Supplement: Supplementary file 1 [file Supplementary_file_1.docx]

**Supplementary Table S1. Overall and Dimension-Specific Psychological Capital Scores Across the Three Latent Profiles**

| **Psychological capital dimensions** | **Total sample (n=327)** | **Low PsyCap profile (n=116)** | **Moderate PsyCap profile (n=131)** | **High PsyCap profile (n=80)** |
| --- | --- | --- | --- | --- |
| Self-efficacy | 4.10 ± 0.74 | 3.41 ± 0.37 | 4.09 ± 0.33 | 5.13 ± 0.36 |
| Resilience | 4.14 ± 0.78 | 3.39 ± 0.39 | 4.18 ± 0.36 | 5.17 ± 0.41 |
| Hope | 4.35 ± 0.64 | 3.76 ± 0.28 | 4.37 ± 0.32 | 5.18 ± 0.43 |
| Optimism | 4.21 ± 0.76 | 3.47 ± 0.34 | 4.27 ± 0.38 | 5.18 ± 0.43 |
| Overall PsyCap score | **4.19 ± 0.69** | **3.50 ± 0.26** | **4.22 ± 0.24** | **5.16 ± 0.32** |

Note:PsyCap scores were calculated as mean scores of the corresponding items. Higher scores indicate higher levels of psychological capital.

**Supplementary Table S2.** Dimension-Specific Model-Estimated Psychological Capital Scores for the Competing Four-Profile Solution

| **Latent class** | **Model-estimated class proportion (%)** | **Self-efficacy** | **Resilience** | **Hope** | **Optimism** |
| --- | --- | --- | --- | --- | --- |
| Class 1 | 37.733 | 3.855 | 3.916 | 4.120 | 3.949 |
| Class 2 | 22.601 | 3.240 | 3.217 | 3.666 | 3.340 |
| Class 3 | 25.663 | 4.556 | 4.607 | 4.715 | 4.693 |
| Class 4 | 14.004 | 5.332 | 5.395 | 5.400 | 5.411 |

Note:Values are dimension-specific means derived from the model-estimated item means; class proportions are model-estimated. The four-profile solution was re-estimated using 2,000 random starts with 500 final-stage optimizations and reproduced the same maximum log-likelihood and substantive class structure. PsyCap, Psychological Capital.

**Supplementary Figure S1. Dimension-Specific Psychological Capital Scores for the Competing Four-Profile Solution.** The y-axis represents dimension-specific means derived from the model-estimated PCQ item means for each latent class, and the x-axis represents the four dimensions of Psychological Capital. Class labels correspond directly to those obtained from the final four-profile Mplus stability run. The y-axis is displayed over the full theoretical 1–7 response range of the PCQ-26.
